# Science big and small

**DOI:** 10.1093/nsr/nwaa239

**Published:** 2020-10-12

**Authors:** Mu-ming Poo

**Affiliations:** Director, CAS Center for Excellence, Brain Science and Intelligence Technology, China

The Fourteenth Five-year Plan is underway for the next phase of China's science and technology development. This will coincide with the inception of several big national projects known as ‘Innovation 2030 Major S&T Projects’. Critical to these major projects is the balance between top-down organized team work and exploratory research by small laboratories. Can big science projects accommodate and benefit from innovative research by small independent laboratories?

Scientists have achieved success in big science projects in the past, from the sequencing of human genome to discoveries of elementary particles and gravitational waves. Recent lunar and Mars explorations are successful examples of big science projects involving a large number of scientists and engineers working together to reach well-defined milestones. Successful big science projects meet the following criteria: First, they have strong and visionary leaders, who are able to attract and organize capable scientists into effective team work. Second, clear and realistic goals and milestones are defined by scientists themselves. Third, the timing for the project is ripe and the field is ready for it.

History has also taught us that many S&T advances originated from discoveries made by small laboratories. For examples, ground-breaking discoveries in biology and biotechnology, such as the structure of DNA, the genetic code, programmed cell death, recombinant DNA, polymerase chain reaction, and stem cell technology—to name a few—were all made by individual scientists through small laboratory-based research in ways that were unpredictable. This raises the concern that big science projects may divert too much resource away from small laboratories, stifling innovative pursuits by individual scientists.

Many Chinese research institutions are filled with small independent laboratories which lack sufficient expertise and resources to address important unsolved problems. Small laboratories could be supported in the past by ostentatiously participating in large team projects, which were viewed as a means of securing funding without serious peer competition and review. Progress were often made by a few leading laboratories of the team, but much funding was distributed to mediocre laboratories packaged into the team via institutional and personal connections. There is concern that Innovation 2030 major projects may fall into the same fate, unless rigorous project management and consequential reviews of the execution and progress of the project are in place.

Big science projects need cutting-edge technologies that require a strong team of innovative researchers. However, such researchers are likely to have their own research interests and ambition for personal achievement, thus are reluctant to become ‘a small screw in a big machine’. Effective resolution of this apparent conflict is critical to big science projects. Thus the project's goal must be enthusiastically supported by all participants, various components of the project should be designed in line with individual scientists’ interest, and mechanisms need to be devised to confer sufficient recognition of individual's contribution to the team work. Most importantly, sufficient resources should also be provided to some innovative scientists who prefer to work alone on subjects that are peripherally related to the project's goal.

Scientific advance is unpredictable even for big science projects, and exploratory efforts by small laboratories on different approaches are essential. While visionary leaders may have a sense of the general trend of science, it is difficult to predict which and when a specific approach will bring the breakthrough. Small laboratories are more effective for interaction among scientists, for inspiring creative ideas, for performing innovative experiments, and for solving intellectually and technically difficult problems. Thus, the ideal setting for a big science project consists of organized groups of small innovative laboratories working towards the same goal, where motivation that drives scientists in their work—personal interest in the particular subject, competition with their peers, and individual recognition by the community—is not diminished by participating in the big project. Such a scenario was indeed present in the Manhattan Project, perhaps the first and most successful big science project.

Importantly, the small laboratory setting is more suitable for training young scientists and establishing mentor-apprentice relationship, in which the lab supervisor directly interacts with lab members and is fully aware of the experimental design, data analysis, and interpretation of results. Students learn ‘the art of soluble’ (to quote Peter Medawar) directly from the mentor through personal interactions that involve not only learning practical skills in solving problems at hand, but also acquiring through permeation the aspiration in science, style and taste in research, and personal integrity when scientific or non-scientific judgments are called for. Such mentor-apprentice relationship has yielded generations of great scientists of the twentieth century. ‘Innovation 2030’ big science projects are winds of our time. With proper organization, they could not only accomplish the set goals, but also sow seeds that will grow and blossom in many fertile fields.

